# Commentary: Determinants of quality of life and treatment satisfaction in dual-diagnosis patients

**DOI:** 10.3389/fpsyt.2023.1173253

**Published:** 2023-05-09

**Authors:** André Wierdsma

**Affiliations:** Department of Psychiatry, Erasmus Medical Center, Rotterdam, Netherlands

**Keywords:** guidelines, strobe, involuntary hospital admission, quality of life, treatment satisfaction

## Introduction

Although psychiatrists and psychologist are familiar with clinical guidelines, awareness of research guidelines can be increased (see https://www.equator-network.org/). Case in point is the reporting by Van Kranenburg et al. on determinants of treatment satisfaction and quality of life in patients in long-term involuntary care (1). To help improve treatment outcomes, this study aimed to identify determinants of treatment satisfaction (assessed with the CAT: Client's Assessment of Treatment scale) and of subjective quality of life (as measured with the MANSA: Manchester Short Assessment of Quality of Life). The authors conclude that being-listened-to and experiencing improvements during treatment are “the most important aspects”. However, study results do not allow this interpretation which could have become apparent simply by using reporting guidelines, in this case STROBE: Strengthening the Reporting of Observational Studies in Epidemiology (2).

### Missings and measurements

First of all, the number of missing values should be reported for all variables of interest and “for each step in the analysis”. The results section shows that 28 patients were non-responders and Table 2 indicates that in the multivariable regression models another 24 missings occurred. Therefore, the total number of patients not included in the final analyses is 52 participants (33%), who may or may not be missing at random. This potential source of bias is not discussed in the limitation section. Next, the STROBE-statement advises authors to report estimated validity or reliability indices “rather than simply citing validation studies”. Yet in the methods section Van Kranenburg et al. refer to previous studies to convey good internal consistency and factorial validity of the CAT and MANSA. Moreover, the authors calculated the mean score of all MANSA-items used and thus failed to address the two-factor structure (life and health-related aspects and quality of living environment), which could account for weak associations (3).

### Problems with *P*-values

Table 1 tests differences in characteristics of responders (*N* = 128) and non-responders (*N* = 28), although there were no real hypotheses to be tested. The STROBE-guideline states that “significance tests should be avoided in descriptive tables.” Based on non-significant *p*-values, Van Kranenburg et al. conclude that “in terms of age, sex, education and diagnosis, non-respondents did not differ from respondents.” However, absence of proof is not proof of absence. In addition, these characteristics are not associated with treatment satisfaction or quality of life, so that in this context the between group similarity is of no consequence.

The STROBE-statement has more on *P*-values: for selecting variables in a multivariable regression analysis “*P*-values are not an appropriate criterion”. And “estimates should be given with confidence intervals.” In contrast, Kranenburg et al. based variable selection on statistical significance and in Tables 3 and 4 only report point estimates of standardized coefficients. As a result, the Helping alliance scale showing a regression coefficient of 0.22 was excluded (*p* = 0.06), whereas the item “I decide whether or not to take medication” indicating perceived coercion was included with univariate coefficient 0.20 (*p* = 0.04). Surely the differences in these regression coefficients and *p*-values are not themselves significant.

### Model fit and interpretation

Finally, with regard to model assumptions and model fit the STROBE-guideline advices to investigate departures from linearity “as it may be wrong to assume a linear relation automatically” and to “give a cautious overall interpretation”. Van Kranenburg et al. do report on multicollinearity between determinants, but other assumptions of linear regression are left unspoken. Model fit R square is estimated at 55% for treatment satisfaction and 29% for quality of life. However, because time of assessment after admission varied widely (between 8 days and 3.5 years), all linear regression models included time of assessment after admission. But the regression coefficient for time of assessment and its contribution to the variance-explained measure are not reported. The authors interpret model variables as “strong independent predictors”, paving way to the so-called Table 2 fallacy, here Tables 3 and 4 (4). Treatment satisfaction and quality of life are regressed on illness insight, justification of admission, and perceived coercion, without taking the indirect or interaction effects of time, insight and attitude into account. And absence of multicollinearity (a characteristic of the sample) is not proof of independence between determinants (a characteristic of the model).

## Discussion

Better understanding of treatment satisfaction and quality of life may help to improve treatment outcomes, but skipping measurement problems, focusing on *p*-values and sifting out some statistically significant variables is not the way forward. Guidelines help to avoid these common pitfalls in research and authors should follow the sound advice from the collaborative work of statisticians, methodologists, epidemiologists, and journal editors. The role of journal editors and reviewers is key to authors' use of the guideline (5). In order to improve the reporting of research and streamline the review process, journals should endorse the STROBE-guideline and the like.

## Author contributions

The author confirms being the sole contributor of this work and has approved it for publication.
